# F-18-fluorodeoxyglucose positron emission tomography-computed tomography for the diagnosis of Takayasu's arteritis in stroke: a case report

**DOI:** 10.1186/1752-1947-2-239

**Published:** 2008-07-24

**Authors:** Carl-Albrecht Haensch, Dirk-Armin Röhlen, Stefan Isenmann

**Affiliations:** 1 Department of Neurology, HELIOS-Klinikum Wuppertal and University of Witten/Herdecke, Heusnerstraße 40, D-42283 Wuppertal, Germany; 2 Radprax Wuppertal, Bergstraße 7-9, D-42105 Wuppertal, Germany

## Abstract

**Introduction:**

Diagnosis of Takayasu's arteritis as the cause of stroke is often delayed because of non-specific clinical presentation. F-18-fluorodeoxyglucose positron emission tomography-computed tomography may help to accurately diagnose and monitor Takayasu's arteritis in stroke patients.

**Case presentation:**

We report the case of a left middle cerebral artery stroke in a 39-year-old man. Laboratory data were consistent with an inflammatory reaction. While abdominal contrast-enhanced computed tomography showed an aneurysm of the infrarenal aorta, only F-18-fluorodeoxyglucose positron emission tomography-computed tomography revealed pathology (that is, intense F-18-fluorodeoxyglucose accumulation) in the carotid arteries, ascending aorta and the abdominal aorta cranial to the aneurysm. After treatment with high-dose prednisone followed by cyclophosphamide, the signs of systemic inflammation decreased and F-18-fluorodeoxyglucose uptake was reduced as compared with the initial scan.

**Conclusion:**

F-18-fluorodeoxyglucose positron emission tomography-computed tomography showed inflammatory activity in the aorta and carotid arteries, suggestive of Takayasu's arteritis in a young stroke patient, and follow-up under immunosuppressive therapy indicated reduced F-18-fluorodeoxyglucose uptake. F-18-fluorodeoxyglucose positron emission tomography-computed tomography appears to be useful in detecting and quantifying the extent of vascular wall activity in systemic large-vessel vasculitis.

## Introduction

Takayasu's arteritis (TA) is a rare, chronic panarteritis localized to the aortic arch or its branches, the ascending thoracic aorta, the abdominal aorta, or the entire aorta. TA, also known as 'pulseless disease', is more common in women than in men. More than half of patients may develop diverse neurological manifestations, such as headache, visual disturbances, seizures, transient ischemic attack, cerebral infarction, intracerebral hemorrhage, and orthostatic syncopal attacks [1]. Typically, early symptoms of systemic inflammatory disease are followed by inflammation of the aorta and its branches. TA has two distinctive phases: a prepulseless (inflammatory or systemic) phase and a pulseless phase. Clinical disease preceding the pulseless phase of TA will not fulfill the diagnostic criteria of the American College of Rheumatologists for TA, which are based on advanced disease [2]. As histological diagnosis is usually impractical, angiography, and, more recently, contrast enhanced computed tomography (CT) and magnetic resonance imaging (MRI) have gained a role in the diagnosis. Laboratory abnormalities may include anemia, leukocytosis, increased erythrocyte sedimentation rate (ESR), elevated C-reactive protein (CRP), and hypergammaglobulinemia. We suggest that abnormal F-18-fluorodeoxyglucose (18F-FDG) accumulation may be useful in diagnosing the early-phase of TA.

## Case presentation

A 39-year-old right-handed man had sudden onset of unsteady gait, right-sided hemihypesthesia and failure of speech. His medical history was significant for arterial hypertension for the past 2 years and a past history of smoking. ESR had been noted to be elevated (55 mm/hour) for the past 18 months, without any known reason. His initial examination was remarkable for dysarthria, discrete right hemiplegia and gait ataxia. The National Institutes of Health Stroke Scale score was 3. Also, marked hypertension (170/90 mmHg) was present on both sides without a blood pressure difference. Radial pulses were not diminished. Pulse was 85 beats per minute.

**Figure 1:**
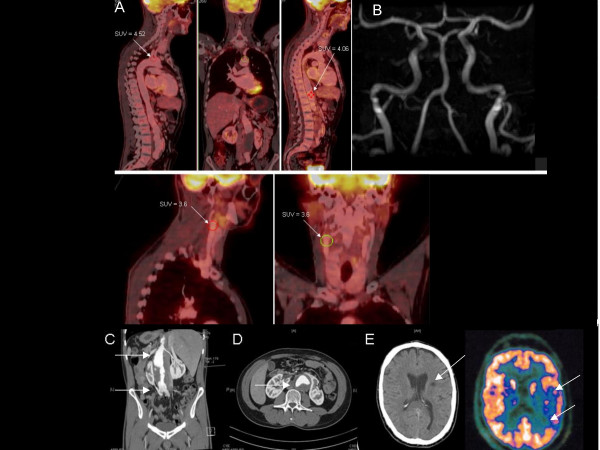
**FDG PET-CT, MR-angiography and CT in a patient with TA and stroke**. (A) F-18-fluorodeoxyglucose positron emission tomography-computed tomography showing fluorodeoxyglucose accumulation in the carotid arteries, ascending aorta, and the abdominal aorta cranial to the aneurysm (arrows with corresponding maximal standardized uptake values). (B) Magnetic resonance angiography of the cerebral vessels. (C), (D) Infrarenal aortic aneurysm (13 cm × 4.8 cm) with mural thrombus (arrows). (E) Computed tomography and fluorodeoxyglucose positron emission tomography revealing middle cerebral artery stroke (arrows).

**Figure 2:**
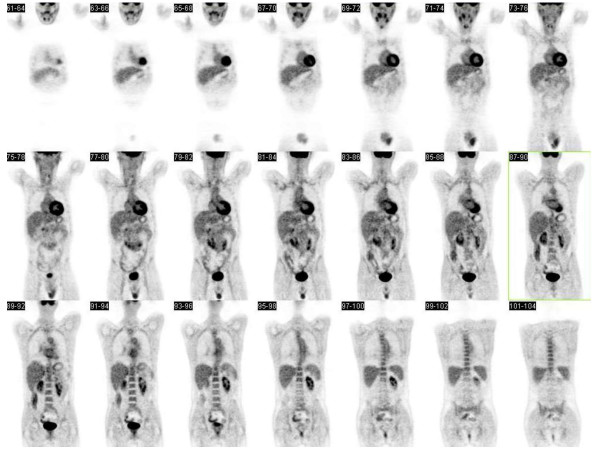
F-18-fluorodeoxyglucose positron emission tomography-computed tomography maximum intensity projection images.

The head CT showed a demarcated infarction in the left middle cerebral artery territory. A subsequent MRI scan also revealed a subacute left-sided pontine infarction. Magnetic resonance angiography demonstrated no abnormalities of the cerebral vessels (Figure 1B). While transcranial and extracranial ultrasound was normal, abdominal CT showed an aneurysm of the infrarenal aorta with a diameter of 4.8 cm and a marginal thrombus (Figure 1C and 1D). CT angiography of the coronary arteries was normal. Transthoracic and transesophageal echocardiography and hypercoagulability state (proteins C and S, factor V Leiden mutation, homocysteine level, and lupus anticoagulant) were unremarkable. Laboratory studies revealed increases in ESR (46 mm/hour), CRP (47 mg/l), C3-complement levels (194 mg/dl) and hypergammaglobulinemia (1.11 g/dl). MPO/P-ANCA, C-ANCA and rheumatoid factors were negative. Cerebrospinal fluid protein level (94 mg/dl) was increased without intrathecal synthesis of immunoglobulins. For suspected arteritis and to detect systemic inflammatory disease, whole body scans in one session on a dual modality positron emission tomography (PET)-computed tomography system were performed 60 minutes after intravenous administration of 243MBq 18F-FDG. PET and CT images were reconstructed in coronal, sagittal, and transverse planes (Figures 1A and 2).

The inflammatory vascular lesion was evaluated using the standardized uptake value (SUV) of 18F-FDG accumulation as an index. F-18-fluorodeoxyglucose positron emission tomography-computed tomography (18F-FDG PET-CT) revealed intense 18F-FDG accumulation in the carotid arteries (maximal SUV = 3.55), ascending aorta (SUV = 4.52), and the abdominal aorta cranial to the aneurysm (maximal SUV = 4.65). No 18F-FDG accumulation was observed in other sites. The patient was given intravenous high-dose bolus prednisone followed by pulse cyclophosphamide, which resulted in a favorable clinical course and normalization of the ESR (4 mm/hour).

**Figure 3:**
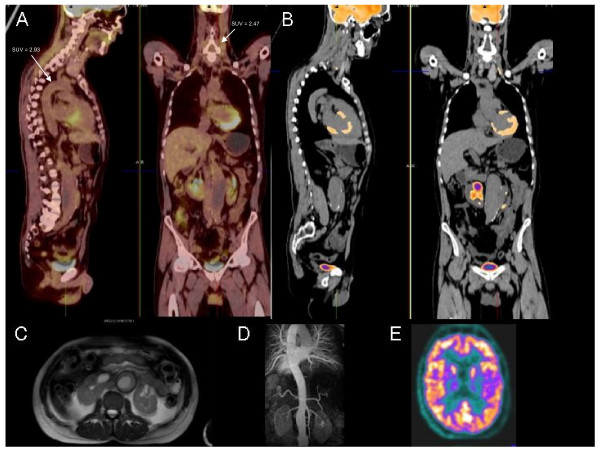
**Follow-up 18F-FDG PET-CT study revealed reduced wall enhancement under immunosuppressive therapy**. (A), (B) Resolution of fluorodeoxyglucose uptake after immunosuppressive treatment. (C), (D) T2-weighted images and magnetic resonance angiography appear normal except for infrarenal aorta aneurysm. (E) Improvement of metabolism in the left temporal region.

In a follow-up 18F-FDG PET-CT study after 2 months the patient revealed reduced wall enhancement in the aorta (maximal SUV = 2.93) and in the left carotid artery (maximal SUV = 2.47) under immunosuppressive therapy. Unchanged increased glucose utilization was found in the right carotid artery (maximal SUV = 3.6). MRI and magnetic resonance angiography showed no abnormalities besides the infrarenal aneurysm at this time (Figure 3).

## Discussion

Vasculitis is reported to be responsible for 3% to 5% of strokes that occur in people under 50 years old. TA typically occurs in patients younger than 50 years old. Central nervous system involvement is seen in up to one-third of cases and is secondary to carotid artery stenosis, cerebral hypoperfusion, and subclavian steal syndrome. We have described the case of a young man in whom stroke was the initial presentation of TA. From a clinical point of view, aortitis most often presents as a vague syndrome of malaise, fever, and weight loss, while blood tests indicate an inflammatory reaction.

18F-FDG PET-CT is an imaging technique that can be used to assess regional differences in glucose metabolism. Inflammatory cells take up increased amounts of glucose, and therefore FDG accumulation on PET-CT scanning has been reported in patients with TA [3-6]. In contrast to other imaging modalities in TA, FDG PET-CT measures inflammation through the metabolic activity in the arterial wall [7]. The cutoff point of maximal SUV for the diagnosis of vascular inflammation was estimated to be 1.3 with a sensitivity for TA of 90.9% and a specificity of 88.8% [8].

## Conclusion

To the best of the authors' knowledge, FDG PET-CT findings have not previously been reported in patients with TA and stroke. Coregistration of 18F-FDG PET and CT showed the anatomical distribution of the inflammation in the walls of the aorta and carotid arteries. This method offers the advantages of early detection of disease activity and the global assessment of the arterial system in a single examination.

## Abbreviations

18F-FDG: F-18-fluorodeoxyglucose; CRP: C-reactive protein; CT: computed tomography; ESR: erythrocyte sedimentation rate; MRI: magnetic resonance imaging; PET: positron emission tomography; SUV: standardized uptake value; TA: Takayasu's arteritis.

## Competing interests

The authors declare that they have no competing interests.

## Authors' contributions

C–AH and SI contributed to the care of the patient, as well as to writing and reviewing the manuscript. D–AR performed the F-18-fluorodeoxyglucose positron emission tomography-computed tomography. All authors read and approved the final manuscript.

## Consent

Written informed consent was obtained from the patient for publication of this case report and any accompanying images. A copy of the written consent is available for review by the Editor-in-Chief of this journal.
